# Comparative Analysis of Impatiens Leaf Transcriptomes Reveal Candidate Genes for Resistance to Downy Mildew Caused by *Plasmopara obducens*

**DOI:** 10.3390/ijms19072057

**Published:** 2018-07-15

**Authors:** Krishna Bhattarai, Weining Wang, Zhe Cao, Zhanao Deng

**Affiliations:** Department of Environmental Horticulture, Gulf Coast Research and Education Center, IFAS, University of Florida, Wimauma, FL 33598, USA; krishnabhattarai@ufl.edu (K.B.); billwang@ufl.edu (W.W.); cjun01@gmail.com (Z.C.)

**Keywords:** candidate disease resistance gene, disease resistance, downy mildew, garden impatiens, leaf transcriptome, New Guinea impatiens, RNA-Seq

## Abstract

Impatiens downy mildew (IDM) is a devastating disease to garden impatiens. A good understanding of IDM resistance in New Guinea impatiens is essential for improving garden impatiens resistance to this disease. The present study was conducted to sequence, assemble, annotate and compare the leaf transcriptomes of two impatiens cultivars differing in resistance to IDM, reveal sequence polymorphisms and identify candidate genes for IDM resistance. RNA-Seq was performed on cultivars Super Elfin^®^ XP Pink (SEP) and SunPatiens^®^ Compact Royal Magenta (SPR). De novo assembly of obtained sequence reads resulted in 121,497 unigenes with an average length of 1156 nucleotides and N50 length of 1778 nucleotides. Searching the non-redundant protein and non-redundant nucleotide, Swiss-Prot, Kyoto Encyclopedia of Genes and Genomes and Clusters of Orthologous Groups and Gene Ontology databases, resulted in annotation of 57.7% to 73.6% of the unigenes. Fifteen unigenes were highly similar to disease resistance genes and more abundant in the IDM-resistant cultivar than in the susceptible cultivar. A total of 22,484 simple sequence repeats (SSRs) and 245,936 and 120,073 single nucleotide polymorphisms (SNPs) were identified from SPR and SEP respectively. The assembled transcripts and unigenes, identified disease resistance genes and SSRs and SNPs sites will be a valuable resource for improving impatiens and its IDM resistance.

## 1. Introduction

The Genus *Impatiens* belongs to the family Balsaminaceae, which consists of about 850 species of mostly succulent annual or perennial herbs [1]. Wild *Impatiens* plants are primarily found in the mountainous regions of South-East Asia, south China, India and Africa while some species are also found in Japan, Europe, Russia and North America [2]. Garden impatiens (*Impatiens walleriana* Hook.f.) is one of the most popular flowers in the world, widely grown in garden beds, borders and woodland gardens as bedding plants and in containers, window boxes and hanging baskets as house plants [3,4]. Impatiens flowers are available virtually in all colors [3,4].

Impatiens downy mildew (IDM), caused by *Plasmopara obducens* (J. *Impatiens noli-tangere*), is a devastating disease to impatiens and can cause rapid defoliation and plant death. This disease was first reported in Germany in 1877 on *Impatiens noli-tangere*, a wild species of *Impatiens* native to many temperate countries in the northern hemisphere [5]. During the 1880s, *P. obducens* was identified in North America in native *Impatiens* species including *I. capensis* (synonym *I. biflora*), *I. fulva* and *I. pallida* [5]. The first recent occurrence of IDM on *I. walleriana* was reported in the UK in 2003 [6]. Subsequently it spread to other countries in Europe (including Norway (2010), Serbia (2011) and Hungary (2011)) and other continents [7,8,9]. IDM was reported in Taiwan and Japan in 2003 and in Australia in 2008 [10,11,12]. Since then, IDM has been problematic in these countries. In the USA, IDM appeared on *I. walleriana* plants in California in 2004 [13]. In 2011, IDM was reported in eleven states in the USA including Massachusetts, New York and Minnesota [14]. By 2012, this disease spread to 33 states [13]. In 2013, IDM was reported in Hawaii [15]. Outbreak of this disease in the USA in this short period of time has caused the loss of hundreds of millions of dollars, reducing the national annual wholesale value of impatiens from approximately $150 million in 2005 to $65 million in 2015 [16]. While *I. walleriana* is highly susceptible to IDM, New Guinea Impatiens (NGI) (*Impatiens hawkeri*) and its interspecific hybrids have shown resistance to IDM [17]. NGI has been grown as a substitute of garden impatiens currently and occupies a $55-million wholesale market [16]. Although the market of NGI is increasing, it ranked third among the alternatives of garden impatiens [18].

There has been a strong interest in introducing IDM resistance into *I*. *walleriana* cultivars due to continued strong consumer demand. *Impatiens walleriana* cultivars are available in a wide array of vibrant colors, possess excellent shade tolerance and are well adapted to a wide range of growing conditions in containers and garden beds [3]. Moreover, the ease of culturing, wide and greater availability of the seeds and cutting materials has increased the demand of garden impatiens [3]. Use of traditional breeding approaches to transferring the resistance from NGI to garden impatiens has been impeded by the differences between NGI and garden impatiens in chromosome number (2*n* = 2*×* = 16 in *I*. *walleriana* and 2*n* = 2*×* = 32 in NGI), size and morphology [19]. Hence, it is necessary to identify and isolate the gene(s) conferring IDM resistance in NGI and transfer the identified gene(s) into garden impatiens.

Disease resistance (*R*) genes are the most important component of the plant defense mechanism to confer resistance to pathogens carrying matching avirulence genes [20]. A great majority of isolated plant *R* genes encode nucleotide-binding site leucine-rich repeats (NB-LRR) proteins [21]. Based on structure, NB-LRR proteins are separated into Toll/interleukin-1 receptor (TIR)-domain-containing (TNL) and coiled coil (CC)-domain-containing (CNL) subfamilies. Plant NB-LRR proteins function in a network through signaling pathways and induce a series of defense responses such as initiation of oxidative burst, calcium and ion fluxes, mitogen-associated protein kinase cascade, induction of pathogenesis-related genes and hypersensitive responses [22,23,24,25]. Recent studies have shown that the TNL subfamily R proteins transduce signals through the enhanced disease susceptibility 1 (EDS1) protein and the CNL subfamily R proteins do so through non-race specific disease resistance 1 (NDR1) protein with some interchanges occurring in different plants [26]. There also exists a separate independent signal transduction pathway activated by Arabidopsis CNL proteins RPP8 and RPP13.

Few genomic resources are available in impatiens. Genomic and transcriptomic data are needed in impatiens to facilitate the identification of gene(s) conferring IDM resistance in NGI and the development of simple sequence repeat (SSR) and single nucleotide polymorphism (SNP) markers for impatiens breeding. With the rapid development of next generation sequencing and analytical tools, whole-genome and transcriptome sequencing has become possible in non-model organisms. Transcriptome sequencing and characterization can provide a descriptive and biological insight of the functionality of an organism. Recently, transcriptome sequencing has been used commonly to identify candidate *R* genes and to develop molecular markers for disease resistance breeding [27,28]. There has been very few or no study on genes involved in disease resistance using transcriptome sequencing in impatiens. Here, we report the sequencing, assembly, annotation and characterization of the leaf transcriptomes of two impatiens cultivars (Super Elfin^®^ XP Pink (SEP) and SunPatiens^®^ Compact Royal Magenta (SPR)) that differ in IDM resistance and the use of the transcriptome data to identify differentially expressed transcripts and candidate *R* genes and SSR and SNP sites for development of molecular markers. Results of this study, including the assembled transcriptomes and the identified candidate genes and polymorphic sites, will greatly facilitate the development of molecular markers for dissecting the resistance mechanism for downy mildew resistance in NGI, tagging the responsible gene loci and expediting the improvement of downy mildew resistance in impatiens.

## 2. Results

### 2.1. HiSeq Sequencing and De Novo Assembly

A total of over 126 million raw reads were generated from the leaf transcriptomes of the two impatiens cultivars, resulting in approximately 117 million clean reads for the two cultivars and approximately 58 million clean reads per cultivar. More than 97% of the reads had a Phred score of 20. The GC content of impatiens sequence reads was approximately 45% (Table 1). De novo assembly of the impatiens leaf transcriptomes in Trinity resulted in 121,497 unigenes containing approximately 140 million bases of nucleotides. The average length of these unigenes was 1156 nucleotides and the N50 was 1778 nucleotides. The total number of contigs for SPR and SEP was 122,166 and 104,752, respectively. These contigs were functionally annotated, which produced 87,415 and 69,369 annotated unigenes for SPR and SEP, respectively (Table 2).

There were 42,789 and 41,853 distinct singletons identified in SPR and SEP, respectively (Table 2). There were 12,835 (14.68%) and 9431 (13.60%) unigenes that were of 2000 or more nucleotides in length in SPR and SEP cultivars (Table S1). Similarly, 22,656 (18.55%) and 22,175 (21.17%) of contigs with more than 500 nucleotides length were assembled in SPR and SEP cultivars respectively (Table S2).

### 2.2. Functional Annotation

Unigenes were annotated by searching against protein databases including the non-redundant protein (NR), Swiss-Prot, Kyoto Encyclopedia of Genes and Genomes (KEGG) and Clusters of Orthologous Groups (COG) using the BLASTx with an *E*-value cut-off of 1 × 10^−5^ and to the nucleotide database NT using the BLASTn with *E*-value cutoff of 1 × 10^−5^. Out of the 121,497 unigenes, 91,187 were annotated (Table 3).

**Figure 1:**
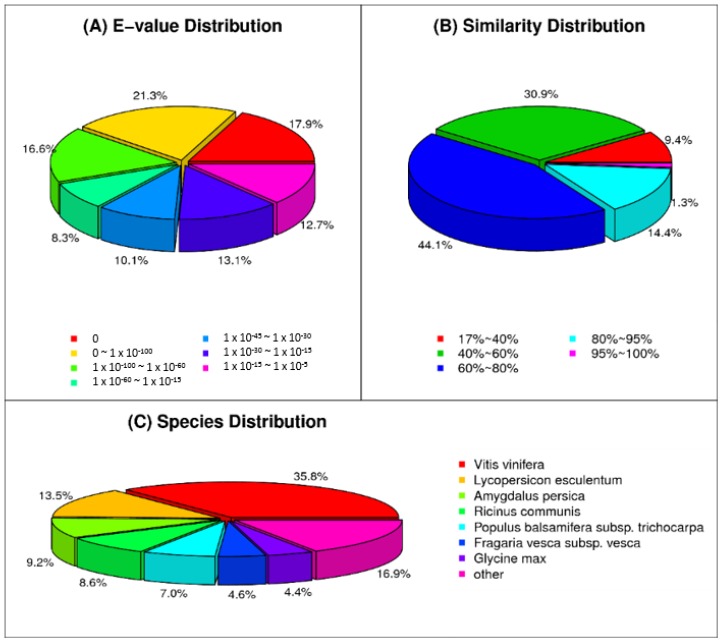
Distribution of similarities of impatiens unigenes with other plant species based on searching the non-redundant protein database according to (**A**) *E*-value (**B**) Similarity and (**C**). In relation to other species with unigenes similarity.

As shown in Table 3, more than 75% of the impatiens unigenes were annotated with at least one of the databases. At the nucleotide level, significant hits (*E*-value 1 × 10^−5^) were observed for 71,482 (58.83%) unigenes in the non-redundant nucleotide database. Comparing the unigenes in the non-redundant protein database, 89,490 (73.66%) unigenes had significant hits (*E*-value 1 × 10^−5^). The similar distribution of impatiens unigenes to other plant species, whose transcriptome sequences and gene annotations are available, show that the impatiens transcriptomes are closely related with *Vitis vinifera* (35.8%), *Lycopersicum esculentum* (13.5%), *Amygdalus persica* (9.2%), *Ricinus communis* (8.6%), *Populus balsamifera* subsp. *trichocarpa* (7.0%)*, Fragaria vesca* subsp. *vesca* (4.6%) and *Glycine max* (4.4%) (Figure 1). Similarly, 59,403 (48.89%), 54,521 (44.87%), 37,576 (30.93%) and 70,190 (57.77%) of the impatiens unigenes had similarity hits in SWISS-Prot, KEGG, COG and GO databases, respectively (Table 3).

### 2.3. COG Classification and KEGG Pathway Mapping

More than 54,521 impatiens unigenes (44%) were annotated in the KEGG database (Table 3). These unigenes were classified into 128 pathways, of which the primary five classes are: cellular processes (3.13%), environmental information processing (2.34%), genetic information processing (16.41%), metabolism (75%) and organismal systems (4%) (Table 4).

**Figure 2:**
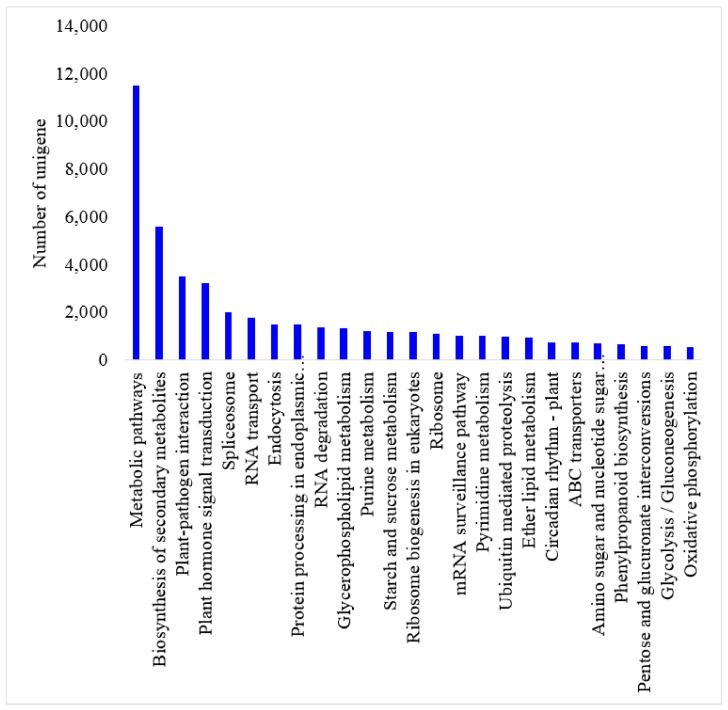
Top 25 pathways to which impatiens leaf transcriptomes were mapped using the Kyoto Encyclopedia of Genes and Genome Database (KEGG).

The top 25 mapped pathways annotated by the KEGG database are shown in Figure 2, among which metabolic pathways (24.70%) was the predominant one, followed by biosynthesis of secondary metabolites (12.01%), plant-pathogen interaction (7.50%), plant hormone signal transduction (6.93%) and spliceosome (4.30%) (Table 5).

**Figure 3:**
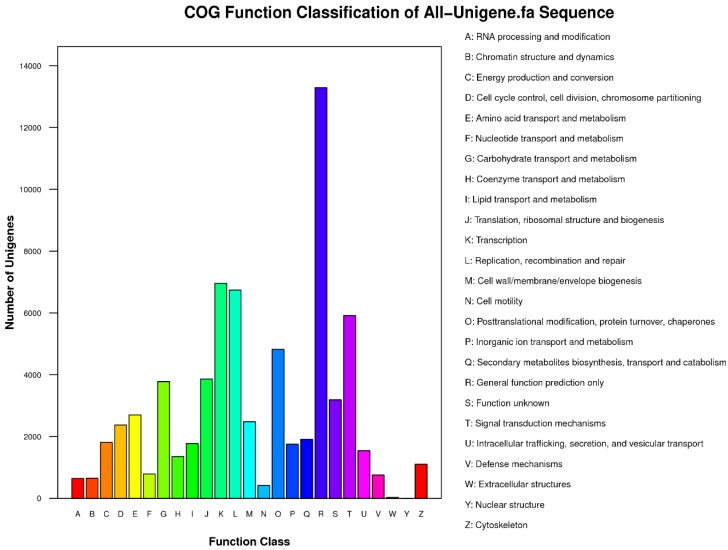
Functional classes of unigenes based on the Cluster of Orthologous Group (COG).

COG classification distributed the annotated impatiens unigenes into 25 categories of functional class. The General Function Prediction Only class contained 13,290 impatiens unigenes, followed by the Transcription class with 6959 impatiens unigenes. The Nuclear Structure class category had only one impatiens unigene (Figure 3). All the categories are shown in Figure 3.

### 2.4. Functional Annotation Based on GO Classification

**Figure 4:**
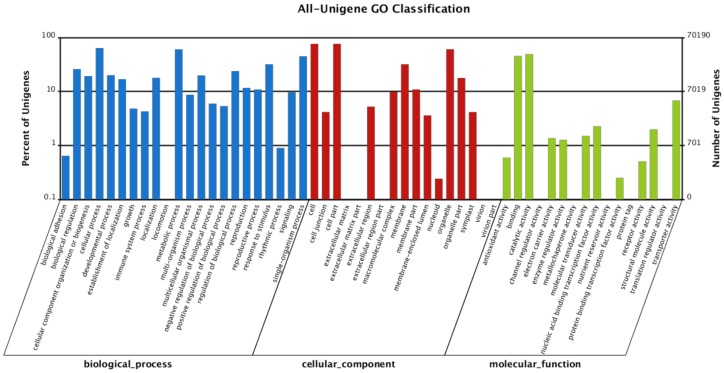
Classification of impatiens unigenes based on the Gene Ontology database.

GO classification differentiated 70,190 impatiens unigenes into at least one of the three categories: biological process, cellular component and molecular function and 55 sub-categories. There were 22 sub-categories in the biological process, among which “cellular process,” “metabolic process” and “single-organism process” had the three highest number of unigenes, involving 44,175, 42,260 and 31,456 unigenes, respectively (Figure 4).

Similarly, in the “cellular component” category, the top three components involving highest number of unigenes were “cell part”, “cell” and “organelle” sub-categories which involved 53,480; 53,480 and 42,491 unigenes, respectively (Figure 4). In the third category “molecular function” the three sub-categories involving the highest number of unigenes were “catalytic activity”, “binding” and “transporter activity” with 34,479; 31,890 and 4764 unigenes, respectively (Figure 4).

### 2.5. Disease Resistance/Defense Genes

In this study, we were particularly interested in genes that are potentially involved in disease resistance and defense-related mechanisms in impatiens. Hence, the impatiens unigenes that were functionally annotated to disease resistance with different databases were selected for further characterization. A total of 164 impatiens unigenes were identified through functional annotation to be potentially involved in disease resistance (Table S3). Fifteen of these unigenes were more than 2000 bp in length each and were found in a greater abundance in the IDM-resistant cultivar SPR than in the IDM-susceptible cultivar SEP (Table 6).

Two of these unigenes were similar to the NB-LRR class *R* genes and another two unigenes were similar to the LRR class *R* genes. In addition, two unigenes showed similarity to the recognition of *Peronospora parasitica 13* (*RPP13*)-like protein 1 family and four unigenes hit putative disease resistance proteins in *Arabidopsis thaliana*. A number of unigenes were similar to the LETM1-like protein, cation efflux family protein, receptor like protein 35, or DNA-binding storekeeper protein-related transcriptional regulator protein classes. There was an uncharacterized unigene, uncharacterized mitochondrial protein AtMg00860 that was found to be involved in the disease resistance mechanism (Table 6).

Sequence similarity comparison of the impatiens unigenes (Table 6) using their coding region sequences and NCBI Blast (https://blast.ncbi.nlm.nih.gov/Blast.cgi) resulted in the separation of these genes into five groups: RPP13, RPM1, RGA, LRR and disease resistance. Three unigenes CL12505.Contig5, CL8803.Contig4, CL8803.Contig5 exhibited similarity to RPP13-like proteins; three unigenes CL10.Contig1, Unigene2713 and Unigene2747 were similar to LRR-proteins; two unigenes CL14259.Contig1 and Unigene13240 similar to RPM1-like proteins; and two unigenes CL1855.Contig4 and Unigene6688 similar to RGA proteins. Some of the unigenes were regulatory proteins like CL14017.Contig1 was similar to zinc transporter like protein, unigene3612 similar to transcription factor mediator associated-protein and CL2138.Contig3 similar to retrotransposon proteins.

The abundance of these unigenes varied between the IDM-resistant cultivar SPR and susceptible cultivar SEP. CL1855.Contig4, similar to *RPP13*-like protein 1 family, reached a FPKM value of 96.38 in SPR but only 0.02 in SEP. Another *RPP13*-like protein 1 family resembling unigene, CL12505.Contig5, had a FPKM value of 2.40 in SPR but 0.45 in SEP. The unigene CL14259.Contig1, similar to CNL *R* genes, reached a FPKM value of 69.99 in SPR but was absent in SEP. Similarly, unigene6688 was quite abundant in SPR (FPKM = 13.23) but absent in SEP and it was similar to TNL *R* genes. CL8803.Contig4 and CL8803.Contig5, both encoding LRR class *R* proteins, were more abundant in SPR (FPKM = 4.60 and 2.61, respectively) than in SEP (FPKM = 0.02). Unigenes CL10.Contig1, CL3381.Contig3, unigene13240 and unigene2747 had a FPKM value of 47.92, 22.67, 24.54 and 37.32 in SRP, respectively, whereas they were present at very low FPKM values or absent in SEP (0.04, 0.06, 0.05 and 0.0, respectively) (Table 6). All these four unigenes were similar to putative *Impatiens noli-tangere*, in *A. thaliana.*

### 2.6. Discovery of SSRs and SNPs

**Figure 5:**
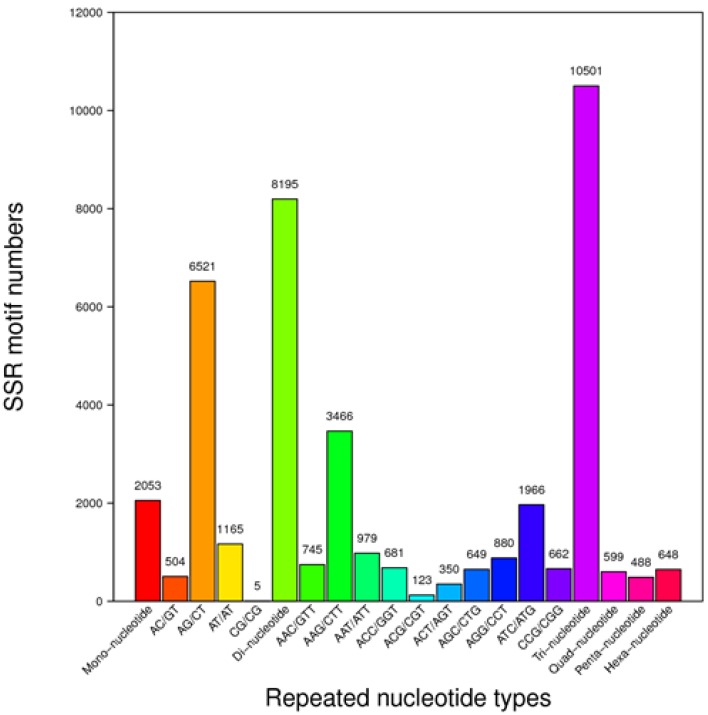
Distribution of simple sequence repeats (SSR) repeat types in impatiens unigenes.

A total of 22,484 SSRs were identified in 19,017 out of the 121,497 impatiens unigene sequences. There were 2986 unigene sequences that each contained more than one SSR and 757 SSRs contained compound repeats. The distribution of SSRs consisted of 2053 mononucleotides, 8195 dinucleotides, 10,501 trinucleotides, 599 tetranucleotides, 488 pentanucleotides and 648 hexanucleotides, among which the trinucleotide type AAG/GTT was the most abundant (3466), followed by the ATC/ATG type (1966) and dinucleotide type AT/AT (1165). The distribution of SSR repeat types among the impatiens unigenes are shown in Figure 5 Primers were developed using the SSRs identified from the unigenes and before and after application of the filtration, there were 44890 and 10389 primers developed (Table S4).

**Figure 6:**
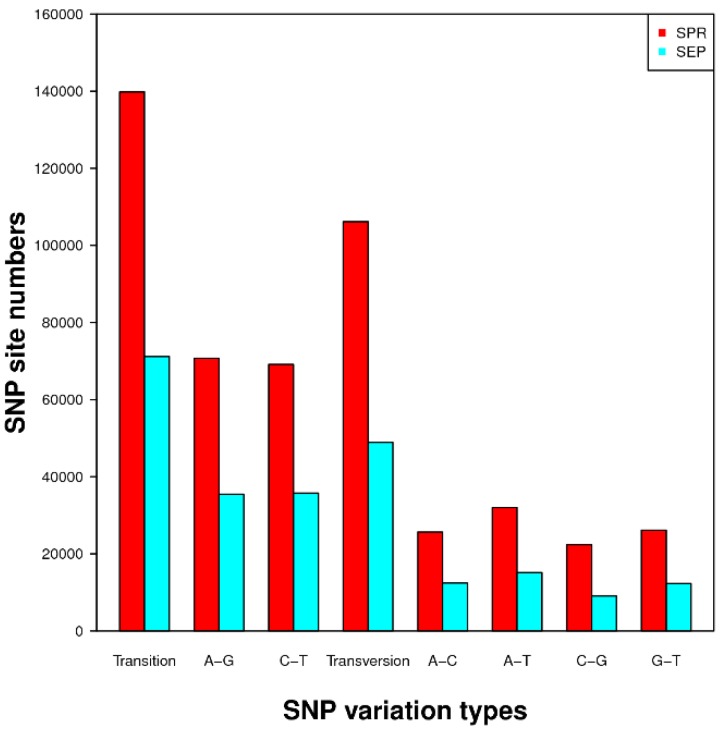
Description and classification of single nucleotide polymorphisms (SNPs) identified in SPR and SEP lines.

The SOAPsnp software was used to identify SNPs in the impatiens transcriptome sequences. The combined assembled impatiens sequence was used as reference and the sequence reads from each cultivar were aligned to the reference individually to call SNPs. SNPs with the number of reads less than seven and the quality score below 20 were discarded. As a result, there were 245,936 and 120,073 SNPs discovered in SPR and SEP cultivars, respectively. The most dominant SNPs type was the transition type, consisting 139,794 (56.84%) and 71,181 (59.28%) for SPR and SEP, respectively, followed by the transversion type consisting of 106,142 (43.16%) and 48,892 (40.72%) for SPR and SEP, respectively. The number of SNPs identified in SPR was higher in all categories and in total in comparison to that of SEP (Figure 6). The list of SNPs in SPR and SEP are supplemented in Tables S5 and S6, respectively. Supplementary Table S7 contain the SNPs, their positions in specific contigs and presence in specific cultivars and supplementary Table S8 contains the SNPs that have been identified in the contigs that are involved in disease resistance and explained in Table 6. 

## 3. Discussion

RNA-Seq has become a very powerful technique for analysis of gene expression, identification of candidate genes involved in the expression of important traits and rapid discovery of large numbers of SSRs and SNPs in non-model plant species [29,30,31]. In this study, the leaf transcriptomes of impatiens were sequenced, assembled, annotated and compared to identify candidate genes potentially involved in resistance against IDM. We found fifteen unigenes that were more than 2000 nucleotides long and had at least 2-fold more transcripts in the IDM-resistant cultivar SPR than in the IDM-susceptible cultivar SEP. Most of the identified candidate genes showed high levels of similarity to the NB-LRR or LRR gene families. Members of the NB-LRR gene family are known to confer plants resistance to diseases caused by bacteria, fungi and viruses and are localized in the cytoplasm [32,33,34,35]. LRR genes have been shown to be involved in fungal resistance. The LRR domain in the NB-LRR and LRR *R*-proteins plays a critical role in defense response by perceiving signals from effectors released by pathogens or pathogens themselves and transducing the signals to initiate the defense responses [36,37,38,39]. The NB-LRR proteins are divided into two groups based on the presence of Toll/interleukin-1 receptor (TIR) or coiled-coil (CC) domain in the amino terminal end. Despite the common function of pathogen recognition, the sequence and signaling mechanism of TNL and CNL proteins are different [40,41,42]. In this study, there were two genes identified as TNLs or CNL encoding proteins.

RPP13 is a part of a NB-ARC domain known for the presence in APAF-1 (apoptopic protease-activating factor-1), R proteins and CED-4 (*Caenorhabditis elegans* death-4 protein) and belongs to the CC-NBS-LRR family that encodes NBS-LRR type R protein with a putative amino-terminal leucine zipper [43]. *RPP13* has been reported to confer resistance to five isolates of *Peronospora parasitica* that caused downy mildew in *A. thaliana* [44]. More than 20 loci for *RPP* genes has been already identified in *A. thaliana* conferring recognition of *P. parasitica* [45]; genes from three loci, *RPP1*, *RPP5* and *RPP8* have been cloned [46,47,48]. *RPP13* has been placed in the subclass of NB-LRR type *R* proteins that have putative leucine zipper (LZ) domain located near the N-terminus [49]. Before it was known that these loci confer resistance to downy mildew in *A. thaliana, RPP13* was only known to possess resistant activity to *Pseudomonas syringae* pathovars [36,37,38,39]. After the understanding of TIR type NB-LRR *R* genes found at the complex *RPP1* and *RPP5* loci [47,48,49]. In *Arabidopsis, RPP13* has been known to be reminiscent of *R* genes *RPM1* [42] and *RPS2* [36,38]. A recent study shows functionally diverged alleles as a distinction in *RPP13* at a simple *R* gene locus [44]. In this study, there were two putative disease resistance RPP13-proteins identified in the IDM-resistant cultivar with higher amount than in the susceptible cultivar. Presence of genes similar to *RPP13* indicate the possibility of *RPP*-like genes conferring the resistance to IDM. Resistance to *P. parasitica* by *RPP13* in *A. thaliana* Niederzenz accession is known to be independent to either Salicylic acid, *NRD1* or *EDS1* [50] triggered by the avirulence gene *ATR13* [51]. *RPP13* encodes protein that is predicted to be located in cytoplasm [43]. In addition to *RPP*-like genes, increased expression of other genes similar to LETM1-like protein, Cation efflux family protein, Receptor like protein 35 and DNA-binding storekeeper protein-related transcriptional regulator indicates the complexity of defense mechanism in the resistant line to IDM. Further research on the mode of resistance in New Guinea Impatiens for IDM could reveal the involvement of these proteins in conjunction with RPP13 to confer resistance or exhibit different mode of action.

Functional annotation of these genes also grouped them into different categories on the basis of similarity. The common method of recognition and signaling mechanisms for resistance exhibited by different classes of gene families differently in unrelated group of pathogens indicates the possibility of functional diversity of *R* genes to defend the host from different kinds of pathogens in different species of host. It has been previously reported that *R* genes exhibit polymorphism regarding the role in defense mechanism. There were other unigenes that were identified to be functionally similar to RPS2-like proteins family in the transcriptome but not all of them were significantly upregulated in the resistant cultivar in this experiment.

Being abundant and randomly distributed in plant genomes, SNPs and SSRs are very useful loci for development of molecular markers for high-density, high-resolution gene or genome mapping and marker-assisted breeding. RNA-Seq and bioinformatics analytical tools have made the discovery of SSR and SNP sites easy and fast. So far, molecular markers have not been reported for downy mildew resistance in impatiens. This study resulted in the discovery of 22,484 SSRs and 245,936 and 120,073 SNPs in SPR and SEP, respectively. These SSR and SNP sites offer a very useful resource for developing molecular markers that can be used by geneticists and breeders for genetic studies and improvement of impatiens.

## 4. Materials and Methods

### 4.1. Plant Materials

IDM-susceptible cultivar Super Elfin^®^ XP Pink (SEP, *I. walleriana*) and IDM-resistant cultivar SunPatiens^®^ (SPR, *I. hawkeri*) were used in this study. Three plants of each cultivar were grown individually in 15-cm plastic containers filled with the commercial soilless potting mixture Faffard^®^ 3B (50% Canadian peat and 50% of the mixture of vermiculite, pine bark and perlite) (Agawam, MA, USA) in a greenhouse facility at the University of Florida’s Gulf Coast Research and Education Center (UF/GCREC), Wimauma, FL, USA. Plants were watered as needed and no fungal pesticides were applied. Mature leaves from both resistant and susceptible plants were sampled and they were instantly frozen in liquid nitrogen and stored in a deep freezer at −80 °C until use.

### 4.2. RNA Isolation, cDNA Synthesis and Sequencing

Frozen leaf tissue samples of SEP and SPR were shipped on dry ice to Beijing Genomics Institute (BGI) in Shenzhen, China. Total RNA was extracted from the impatiens leaf tissues using pBiozol Total RNA Extraction Reagent (BioFlux, Hangzhou, China) following manufacturer’s instructions. The RNA concentration, RNA integrity number (RIN) and the 28S/18S ratio were determined on an Agilent 2100 Bioanalyzer (Agilent Technologies, Santa Clara, CA, USA). An equal amount of RNA from each of the three biological replicates was combined to form a pool of resistant and susceptible RNA samples for mRNA isolation and cDNA synthesis. The combined RNA samples were treated with DNase I to remove residual genomic DNA and mRNA was isolated using magnetic beads with Oligo (dT). The isolated mRNA molecules were fragmented using the Elute Prime Fragment Mix from Illumina TruSeq^TM^ RNA sample prep kit v2 (Illumina, San Diego, CA, USA) at 94 °C for 8 min. The fragmented mRNAs were used as templates for cDNA synthesis. First strand cDNA was synthesized by using the First Strand Master Mix and SuperScript II from Invitrogen (Carlsbad, CA, USA) and amplified at 25 °C for 10 min, 42 °C for 50 min and 70 °C for 15 min. Second cDNA strand was synthesized using the Second Strand Master Mix from Invitrogen at 16 °C for 1 h. The resulted cDNA fragments were purified, their ends were repaired using the End Repair Master Mix from Invitrogen at 30 °C for 30 min and the Ampure XP beads (Beckman Coulter, Brea, CA, USA) and a single adenine (A) base were added to each end of the cDNA fragments. End-repaired and A-added short fragments were joined with adapters. The adapter-ligated cDNA was PCR-amplified to generate sequencing libraries. The libraries were analyzed with an Agilent 2100 Bioanalyzer and the ABI StepOnePlus Real-Time PCR System for quantifying and qualifying the sample library. Sequencing of cDNA was performed on an Illumina HiSeq™ 2000, generating 100 bp paired-end reads.

### 4.3. Sequence Filtering, De Novo Assembly and Transcriptome Analysis

Raw sequence reads were cleaned by removing the adapters attached at the ends and reads with unknown nucleotides more than 5% were removed using BGI’s internal software (filter_fq). Low quality reads with more than 20% of quality value < 10 were removed, Q20 percentage, proportion of unknown nucleotides in clean reads (N) percentage and GC percentage among total nucleotides were calculated and the remaining clean reads were used in further analysis. De novo transcriptome assembly was done using the Trinity software (http://trinityrnaseq.sourceforge.net/) [52] with the following parameters: minimum contig length of 100, minimum glue 3, group pair distance 250, path reinforcement distance 85 and minimum kmer coverage 3 using the cleaned short reads. The longest, non-redundant, unique transcripts were defined as unigenes. Further processing of the sequences for redundancy removal and splicing was done using the sequence clustering software, TIGR Gene Indices Clustering Tools (TGICL) v2.1 (http://sourceforge.net/projects/tgicl/files/tgicl%20v2.1/) with minimum overlap length 40, base quality cutoff of clipping 10 and maximum length of unmatched overhangs 20. Homologous transcript clustering was done using Phrap release 23.0 (http://www.phrap.org/) with repeat stringency 0.95, minimum match 35 and minimum score 35.

Functional annotation of the unigenes was done by comparing them using the NR (non-redundant protein) and NT (non-redundant nucleotide) databases of National Center for Biotechnology Information (http://blast.ncbi.nlm.nih.gov/Blast.cgi), SWISS-PROT database (European Bioinformatics Institute, ftp://ftp.ebi.ac.uk/pub/databases/swissprot/), KEGG (Kyoto Encyclopedia of Genes and Genomes database), COG (Clusters of Orthologous Groups of proteins database) and GO (Gene Ontology) with BLASTx using *E*-value cutoff of 1 × 10^−5^. The best aligning results were used to determine the sequence direction of unigenes. When the results of databases conflicted each other, a priority order of NR, SWISS-PROT, KEGG and COG was followed to determine the sequence direction. When unigenes were found to be unaligned with any of the databases, ESTScan was used to determine the sequence direction [53]. Blast2Go (http://www.blast2go.com/b2ghome) was used to annotate unigenes generated by NR annotation to get GO annotation [54]. After GO annotation, WEGO software was used for functional classification of the unigenes and to understand the distribution of gene functions from the macro level [55]. KEGG database was used for metabolic pathway analysis of gene products in cell and function of gene products. Further study of unigene pathways was done using Path_finder (http://www.genome.jp/) with default parameters [56].

### 4.4. Unigenes Sequence Comparison and Phylogenetic Analysis

The sequences of selected unigenes from impatiens were compared to the Arabidopsis Information Resource (TAIR) Database (https://www.arabidopsis.org/) [57] to show the functional similarity and the best aligning results were used. BLAST was done using CDS sequences of fifteen unigenes (Table 6) involved in disease resistance to the nr/nt database for sequence similarity. 

### 4.5. Identification of SSRs and SNPs

SSRs in the unigene sequences were detected using Microsatellite (MISA) (http://pgrc.ipk-gatersleben.de/misa/misa.html). The minimum cut-off values for the identification of mono-, di-, tri, tetra-, penta- and hexa-nucleotide SSRs were 12, 6, 5, 5, 4 and 4, respectively. Only SSRs with 150-bp flanking sequences on both ends in the unigenes were retained. SSR primers were designed using the Primer3 software (Release 2.3.4, http://www.onlinedown.net/soft/51549.htm) and the default parameters [58]. The SOAPsnp software Release 1.03 (Short Oligonucleotide Analysis Package single nucleotide polymorphism) (http://soap.genomics.org.cn/soapsnp.html) was used to identify SNPs by aligning consensus sequences from each cultivar to the transcriptome assembly [59].

## 5. Conclusions

This study represents the first application of RNA-Seq and sequence analysis to the leaf transcriptomes of garden impatiens, a very important floriculture crop and a very popular garden plant in the world and a representative species of the family Balsaminaceae. The leaf transcriptome sequences will become an invaluable resource for understanding the genetic makeup of this important plant. The transcripts and unigenes assembled can provide valuable template sequences for gene mining and gene expression analysis in impatiens. The unigenes that were abundantly expressed in IDM-resistant impatiens while absent or expressed at very low levels in the IDM-susceptible impatiens and show strong similarity to plant *R* genes can serve as candidate genes for understanding the genetic basis of the IDM resistance in *I*. *hawkeri* and over-expressing in IDM-susceptible *I*. *walleriana* for improved IDM resistance. The SSR and SNP sites identified in the impatiens transcriptomes will be very useful for molecular marker development, gene and genome mapping and identification of specific markers for important plant, foliar and flower traits in impatiens.

## Figures and Tables

**Table 1:** Summary of HiSeq sequencing data for two impatiens leaf transcriptomes.

Samples	Total Raw Reads	Total Clean Reads	Total Clean Nucleotides (Mb)	Q20 (%) ^1^	N (%) ^2^	GC (%)
SunPatiens^®^ Compact Royal Magenta (SPR)	63,769,252	58,266,032	5826	97.59	0.01	44.79
Super Elfin^®^ XP Pink (SEP)	62,996,124	58,834,368	5883	97.50	0.01	45.11
Total	126,765,376	117,100,400	11,710			

^1^ Q20 (%) is the proportion of nucleotides with Q20 quality value greater than 20. ^2^ N (%) is the proportion of unknown nucleotides in clean reads.

**Table 2:** Description of the impatiens transcriptome assembly and quality of unigenes and contigs.

Types	Sample	Total Number	Total Length (nt)	Mean Length (nt)	N50	Total Consensus Sequences	Distinct Clusters	Distinct Singletons
Contig	SunPatiens^®^ Compact Royal Magenta (SPR)	122,166	48,323,299	396	965			
	Super Elfin^®^ XP Pink (SEP)	104,752	44,944,832	429	1052			
Unigene	SunPatiens^®^ Compact Royal Magenta (SPR)	87,415	88,434,291	1012	1774	87,415	44,629	42,789
	Super Elfin^®^ XP Pink (SEP)	69,369	67,514,711	973	1726	69,369	27,516	41,853
	All	121,497	140,506,651	1156	1778	121,497	78,448	43,049

**Table 3:** Annotation of impatiens unigenes using the NT (the non-redundant nucleotide database), NR (the non-redundant protein database), SWISS-PROT, COG (Clusters of Orthologous Groups of proteins), KEGG (Kyoto Encyclopedia of Genes and Genomes) and GO (Gene Ontology Consortium) databases.

Database	Unigene Annotated	Percentage
NR	89,490	73.66
NT	71,482	58.83
Swiss-Prot	59,403	48.89
KEGG	54,521	44.87
COG	37,576	30.93
GO	70,190	57.77
Total annotated genes	91,187	75.05
Total unigenes	121,497	100.00

**Table 4:** Classification of annotated unigene pathways according to categorical processes based on KEGG Pathway Mapping.

Pathway Category	Number	Percentage
Cellular Processes	4	3.13
Environmental Information Processing	3	2.34
Genetic Information Processing	21	16.41
Metabolism	96	75.00
Organismal Systems	4	3.13
Total	128	100

**Table 5:** Number of impatiens unigenes involved in top 25 pathways listed by KEGG database.

Pathway	Genes with Pathway Annotation	% of Genes
Metabolic pathways	11,493	24.70
Biosynthesis of secondary metabolites	5590	12.01
Plant-pathogen interaction	3491	7.50
Plant hormone signal transduction	3224	6.93
Spliceosome	2001	4.30
RNA transport	1760	3.78
Endocytosis	1488	3.20
Protein processing in endoplasmic reticulum	1484	3.19
RNA degradation	1366	2.94
Glycerophospholipid metabolism	1334	2.87
Purine metabolism	1237	2.66
Starch and sucrose metabolism	1186	2.55
Ribosome biogenesis in eukaryotes	1171	2.52
Ribosome	1117	2.40
mRNA surveillance pathway	1033	2.22
Pyrimidine metabolism	1027	2.21
Ubiquitin mediated proteolysis	967	2.08
Ether lipid metabolism	943	2.03
Circadian rhythm–plant	764	1.64
ABC transporters	758	1.63
Amino sugar and nucleotide sugar metabolism	712	1.53
Phenylpropanoid biosynthesis	660	1.42
Pentose and glucoronate interconversions	585	1.26
Glycolysis / Gluconeogenesis	584	1.25
Oxidative phosphorylation	564	1.21
Total	46,539	100

**Table 6:** List of impatiens unigenes selected based on more than 2-fold higher abundancy in IDM-resistant impatiens (SPR) compared to IDM-susceptible impatiens (SEP) that were annotated to previously known genes for disease resistance or defense using NR (non-redundant protein), NT (non-redundant nucleotide), SWISS-PROT, KEGG (Kyoto Encyclopedia of Genes and Genomes), COG (Clusters of Orthologous Groups of proteins) and GO (Gene Ontology) databases.

Unigene ID	Length (bp)	Abundance (FPKM ^1^) in SPR	Abundance (FPKM ^1^) in SEP	*p*-Value	FDR	Score	Annotation
CL10.Contig1	5073	47.92	0.04	7.13 × 10^−24^	2.66 × 10^−23^	164	Putative disease resistance protein At5g05400
CL12505.Contig5	4428	2.40	0.45	4.91 × 10^−29^	2.04 × 10^−28^	689	Putative disease resistance RPP13-like protein 1
CL12796.Contig1	2489	9.11	0.38	8.20 × 10^−108^	7.28 × 10^−107^	103	LETM1-like protein
CL14017.Contig1	2600	18.20	0.95	2.35 × 10^−212^	3.47 × 10^−211^	805	Cation efflux family protein
CL14259.Contig1	2360	69.99	0.00	0	0	400	Disease resistance protein (CC-NB-LRR class) family
CL1855.Contig4	2257	96.38	0.02	5.25 × 10^−09^	1.17 × 10^−08^	473	Putative disease resistance RPP13-like protein 1
CL2138.Contig3	2505	5.82	0.00	1.69 × 10^−88^	1.32 × 10^−87^	75.5	Uncharacterized mitochondrial protein AtMg00860
CL3381.Contig3	4495	22.67	0.06	0	0	543	Probable disease resistance protein At5g45510
CL8803.Contig4	2187	4.60	0.02	2.46 × 10^−59^	1.51 × 10^−58^	54.7	Leucine-rich repeat (LRR) family protein
CL8803.Contig5	2321	2.61	0.02	1.99 × 10^−35^	9.18 × 10^−35^	73.2	Leucine-rich repeat (LRR) family protein
Unigene2713	2869	7.12	0.00	5.52 × 10^−94^	4.49 × 10^−93^	254	Receptor-like protein 35
Unigene13240	2592	24.54	0.05	1.00 × 10^−123^	9.81 × 10^−123^	414	Putative disease resistance protein At1g59780
Unigene2747	2575	37.32	0.00	2.08 × 10^−23^	7.68 × 10^−23^	308	Putative disease resistance protein At3g14460
Unigene3612	3233	2.05	0.04	0.000496	0.000733	177	DNA-binding storekeeper protein-related transcriptional regulator
Unigene6688	2671	13.23	0.00				disease resistance protein (TIR-NB-LRR class)

^1^ FPKM: Fragments per kilobase of transcripts per million mapped reads.

## Supplementary Materials

Supplementary materials can be found at http://www.mdpi.com/1422-0067/19/7/2057/s1. Raw sequence reads of the reported impatiens leaf transcriptomes for SPR and SEP have been deposited in the Sequence Read Archive (SRA, http://www.ncbi.nlm.nih.gov/Traces/sra/) under BioSample accession number SAMN09394245 and SAMN09393910 respectively.
