# Supplementary material for: Comparative Analysis of Impatiens Leaf Transcriptomes Reveal Candidate Genes for Resistance to Downy Mildew Caused by Plasmopara obducens
Source: Int J Mol Sci. 2018 Jul 15;19(7):2057. doi: 10.3390/ijms19072057 (PMC6073305; doi:10.3390/ijms19072057)
Supplement: Supplementary file 1 [file ijms-19-02057-s001.zip › List of supplymentary files.pdf]

Table S1: Length distribution of unigenes of cultivars Super Elfin® XP Pink (SEP) and SunPatiens® Compact Royal Magenta (SPR)

Table S2: Length distribution of contigs of cultivars Super Elfin® XP Pink (SEP) and SunPatiens® Compact Royal Magenta (SPR)

Table S3: Differentially expressed disease resistance genes

Table S4: Single Sequence Repeat primers

Table S5: Single nucleotide polymorphisms (SNPs) in garden impatiens, Super Elfin XP Pink (SEP) cultivar

Table 76: Single nucleotide polymorphisms (SNPs) in New Guinea impatiens, Sun Patiens Compact Royal Magenta (SPR) cultivar

Table S7: List of single nucleotide polymorphisms (SNPS) with their position and presence in specific cultivars of impatiens

Table S8: List of single nucleotide polymorphisms (SNPs) identified in the unigenes that are putatively involved in disease resistance in Impatiens
